# An LRPPRC-HAPSTR1-PSMD14 interaction regulates tumor progression in ovarian cancer

**DOI:** 10.18632/aging.205713

**Published:** 2024-04-18

**Authors:** Dongxiao Li, Min Wang

**Affiliations:** 1 Department of Obstetrics and Gynecology, Shengjing Hospital of China Medical University, Shenyang, China

**Keywords:** HAPSTR1, LRPPRC, ovarian cancer, PSMD14

## Abstract

Ovarian cancer is the second most common cause of gynecologic cancer death. Chemoresistance and metastasis remain major challenges for current treatment. Previously, *HAPSTR1* was shown to be a target gene of a paclitaxel resistance-associated miRNA. However, the biological function and underlying molecular mechanisms of HAPSTR1 in ovarian cancer progression remain unclear. Herein, we aimed to measure HAPSTR1 expression in ovarian cancer specimens and examine its correlations with clinical features and key functional interactions with other genes and proteins. An immunohistochemistry assay showed that HAPSTR1 was overexpressed in ovarian cancer tissues and was significantly associated with the FIGO stage and clinical outcome. HAPSTR1 overexpression promoted proliferation, invasion and migration in cellular and mouse models, whereas inhibition induced the opposite effects. In addition, HAPSTR1 stimulated the EMT pathway and affected the expression of autophagy biomarkers. Mechanistically, we demonstrated that HAPSTR1 is bound to LRPPRC and PSMD14 via immunoprecipitation. HAPSTR1 suppressed LRPPRC ubiquitination and recruited PSMD14 to interact with LRPPRC. Moreover, LRPPRC knockdown reversed HAPSTR1-mediated improvement in cellular proliferation, invasion, and migration. Our study is the first detailed and comprehensive analysis of HAPSTR1 in cancer progression and offers an experimental basis for the clinical treatment of ovarian carcinoma.

## INTRODUCTION

Ovarian cancer (OV) is the seventh most common cancer and the second most common cause of gynecologic cancer death [1]. Owing to a lack of specific symptoms, only 15% of OVs are diagnosed at an early stage [2, 3]. Despite various attempts to improve the diagnosis and therapy of patients with OV, chemoresistance and metastasis remain major challenges for current treatment [4]. Hence, understanding the mechanisms underlying the progression and metastasis of OV is critical to developing novel therapies [5].

miRNAs are widely appreciated as pervasive regulators of chemoresistance and metastasis [6]. Our group reported miR-134 which was associated with paclitaxel resistance in OV and its target genes [7, 8]. Among these targets, we are particularly interested in C16ORF72. Although the effects of C16ORF72 on cancer cell proliferation and migration have been previously reported [9, 10], the underlying function and mechanism of action of C16ORF72 in OV development remain unclear. *C16ORF72* regulates telomere integrity and p53 [11], A recent study identified C16ORF72 as a HUWE1 substrate and interacting protein that plays an important role in an integrated network of stress response pathways; C16ORF72 was thus renamed HAPSTR1 (HUWE1-Associated Protein modifying STress Responses) [9]. HAPSTR1 is essential for HUWE1 nuclear localization and nuclear substrate targeting [10]. Because HUWE1 and miR-134 affect OV progression [12], we speculate that HAPSTR1, as their biomedically relevant target, may also play a functional role in OV.

Recent studies have illustrated the importance of ubiquitination in regulating HAPSTR1 protein activity and stability [10]. Ubiquitination is a reversible post-translational modification (PTM) process and can be reversed by cleaving Ub from the substrate protein to terminate the signal [13]. PSMD14 is a subunit of the proteasome regulatory particle, where it acts as an intrinsic deubiquitinase, removing polyubiquitin chains from substrate proteins [14]. PSMD14 affects OV progression and inhibits the ubiquitination of LRPPRC and PKM2 [15, 16]. According to recent reports, LRPPRC can affect proliferation, apoptosis, stemness, and autophagy in ovarian cancer [16, 17]. However, the relationship between HAPSTR1, PSMD14 and LRPPRC still remains to be elucidated.

Thus, in this study, we aimed to measure HAPSTR1 expression in OV specimens, examine correlations between its expression and clinical features, and determine key functional interactions with other genes and proteins for a mechanistic study.

## RESULTS

### HAPSTR1 overexpression in OV is correlated with worse clinical outcome

**Figure 1:**
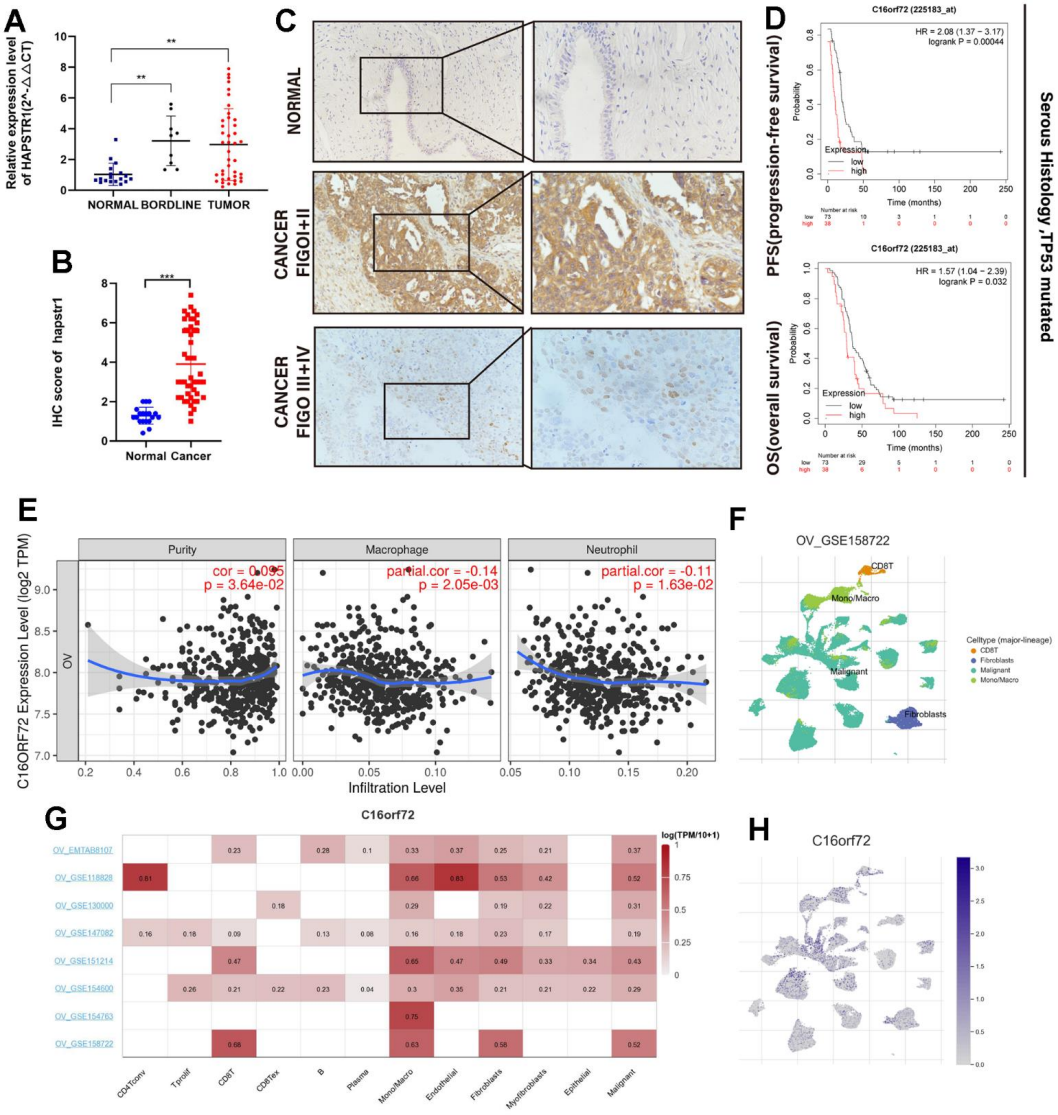
**HAPSTR1 was overexpressed in ovarian cancer tissues and closely related to clinical outcome.** (**A**) Clinical samples in Cohort 1 were used to detect mRNA expression levels of HAPSTR1, including 18 normal ovarian tissues, 9 borderline ovarian tumors, and 40 primary ovarian cancer specimens. (**B**) An immunohistochemistry assay was used to detect protein expression levels of HAPSTR1 in Cohort 2, including 47 epithelial ovarian cancer and 19 normal ovarian tissues. (**C**) IHC results of Cohort 2 showed HAPSTR1 expression was related to FIGO stage. Original magnification, 200X, 400X. (**D**) Kaplan–Meier survival analysis of overall and progression-free survival in patients with ovarian cancer. (**E**) The relationship between HAPSTR1 expression and infiltration levels in the TIMER database. (**F**–**H**) Results from the TISCH database showed a relationship between HAPSTR1 expression levels and immune cells. Each experiment was repeated with three independent replicates. *, *p* < 0.05; **, *p* < 0.01; ***, *p* < 0.001.

The real-time PCR assay of cohort 1 showed that HAPSTR1 expression levels were higher in borderline ovarian tumors and OV tissues than those in normal ovaries (Figure 1A). Statistical analysis of IHC results in cohort 2 also showed a stronger positive staining signal of HAPSTR1 protein in OV samples than in normal ovaries (Figure 1B). Statistical analysis of clinical characteristics showed that in cohort 2, HAPSTR1 expression levels were significantly associated with FIGO stage (Table 1 and Figure 1C). Kaplan–Meier plot analysis indicated that HAPSTR1 expression was significantly associated with short overall and progression-free survival (Figure 1D). There is a significant correlation between the expression level of HAPSTR1 and race (Supplementary Table 1). We also investigated the role of HAPSTR1 in the OV tumor immune environment. The results from the TIMER database showed a negative correlation between HAPSTR1 expression and macrophage (R = -0.14, *p* = 2.05e-03) /and neutrophil (R =-0.11, *p* = 1.63e-02) infiltration in OV (Figure 1F). We used several GEO datasets from the TISCH database to assess the relationship between HAPSTR1 expression and immune cells. The results showed that fibroblasts, mono-/macrocells, and malignant cells were closely associated with HAPSTR1 expression level (Figure 1G). In OV_GSE158722, only four types of immune cells, namely, CD8T, mono/macro, malignant, and fibroblasts, were significantly enriched. Malignant cells exhibited the highest HAPSTR1 expression levels, and mono-/macrocells and fibroblasts also showed a certain degree of enrichment (Figure 1H).

**Table 1:** Relationships between HAPSTR1 expression and clinical characteristics.

**Characteristics**	**Total (n)**	**HAPSTR1 expression (IHC)**		***p-*value**
		**Low**	**High**	
n	44	23	21	
Age (years)				0.460
≥50	29	14 (31.8%)	15 (34.1%)	
<50	15	9 (20.5%)	6 (13.6%)	
FIGO Stage				0.035
III, IV	28	18 (40.9%)	10 (22.7%)	
I, II	16	5 (11.4%)	11 (25%)	
Peritoneal metastasis				0.241
YES	29	17 (38.6%)	12 (27.3%)	
NO	15	6 (13.6%)	9 (20.5%)	
Lymph node metastasis				0.276
YES	14	9 (20.5%)	5 (11.4%)	
NO	30	14 (31.8%)	16 (36.4%)	
CA125 (0-35U/mL)				0.536
NO	32	18 (41.9%)	14 (32.6%)	
YES	11	5 (11.6%)	6 (14%)	
CA199 (0-37U/mL)				1.000
YES	35	20 (51.3%)	15 (38.5%)	
NO	4	2 (5.1%)	2 (5.1%)	
HE4 (<140pmol/L)				0.747
NO	27	14 (34.1%)	13 (31.7%)	
YES	14	8 (19.5%)	6 (14.6%)	
AFP (0-9ng/mL)				1.000
YES	30	17 (51.5%)	13 (39.4%)	
NO	3	2 (6.1%)	1 (3%)	
ROMA-BEFOR (>11.4%)				0.593
YES	33	18 (50%)	15 (41.7%)	
NO	3	1 (2.8%)	2 (5.6%)	
ROMA-AFTER (>29.9%)				0.684
YES	29	16 (44.4%)	13 (36.1%)	
NO	7	3 (8.3%)	4 (11.1%)	
CEA (0-5ng/mL)				1.000
YES	37	19 (46.3%)	18 (43.9%)	
NO	4	2 (4.9%)	2 (4.9%)	
CA-724 (0-6.9U/mL)				0.647
NO	19	9 (22%)	10 (24.4%)	
YES	22	12 (29.3%)	10 (24.4%)	
Menopause				0.437
YES	20	9 (22%)	11 (26.8%)	
NO	21	12 (29.3%)	9 (22%)	

### Functional enrichment analysis of HAPSTR1 and its correlated molecules and experimental verification

**Figure 2:**
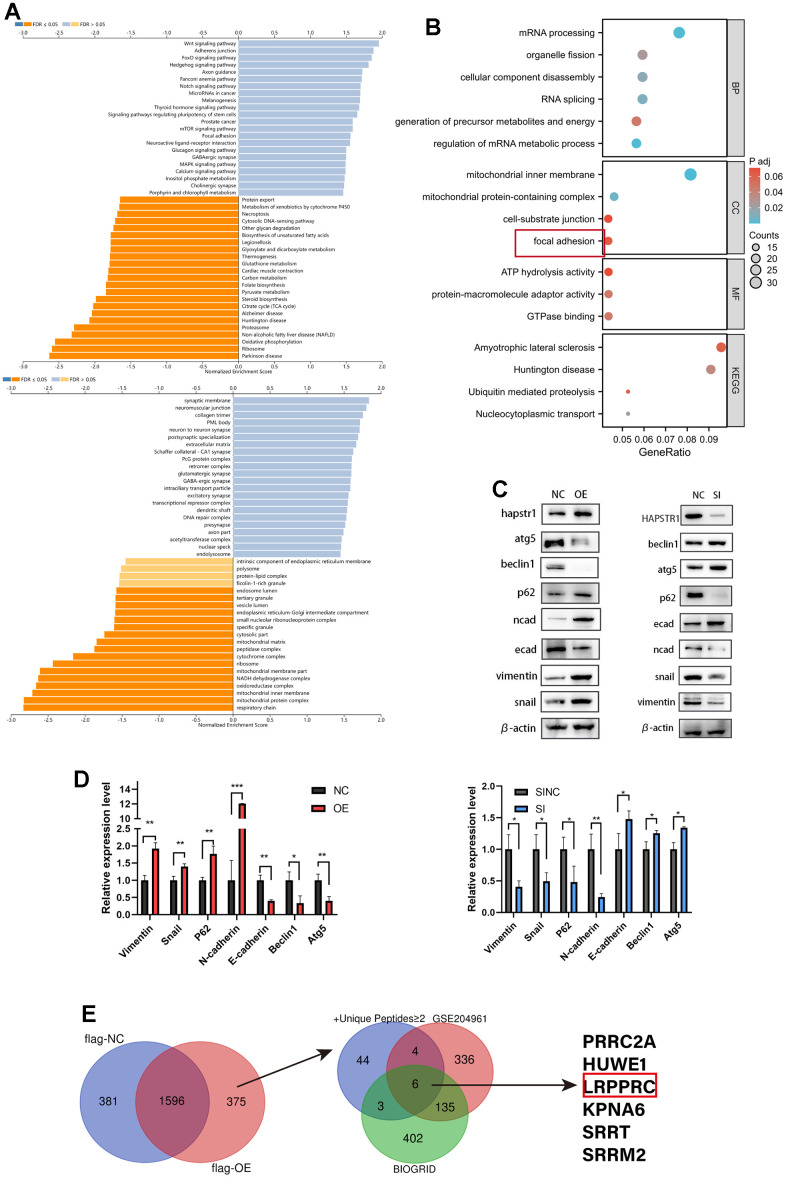
**Functional enrichment analysis of HAPSTR1 and its correlated molecules and experimental verification.** (**A**) GO/KEGG analyses of genes positively and negatively correlated with HAPSTR1 in the LinkOmics database. (**B**) GO/KEGG analysis was used to annotate the results of mass spectrometry (MS). (**C**, **D**) Western blotting demonstrated that HAPSTR1 stimulated the EMT pathway and suppressed autophagy. (**E**) Intersection of data from Immunoprecipitation-Mass Spectrometry (IP-MS), GSE204961, and BIOGRID datasets. *, *p* < 0.05; **, *p* < 0.01; ***, *p* < 0.001.

We used LinkedOmics to investigate genes co-expressed with HAPSTR1 in patient data of OV from TCGA (Supplementary Figure 1A, 1B). GO/KEGG analyses of these positively and negatively related genes indicated that HAPSTR1 plays an important role in the mitochondrial inner membrane, mitochondrial protein complex, respiratory chain, Wnt signaling pathway, adherens junction, and focal adhesion with using Gene Set Enrichment Analysis method (Figure 2A). To examine potential HAPSTR1-interacting proteins, we transfected SKOV3 cells with FLAG-HAPSTR1 plasmids and used anti-FLAG beads to carry out co-immunoprecipitation (COIP) assays. Mass spectrometry (MS) analysis was performed. To confirm the abovementioned functions of HAPSTR1 and its related genes, the MS results were subjected to GO and KEGG enrichment analyses (Figure 2B and Supplementary Tables 2, 3). The results showed that HAPSTR1 and its co-immunoprecipitated proteins were closely associated with focal adhesion. Since focal adhesion is closely related with epithelial-to-mesenchymal transition (EMT), we inferred that HAPSTR1 may also stimulate the EMT pathway. Additionally, mitochondrial function is often associated with autophagy, suggesting that HAPSTR1 affects autophagy. To test our hypothesis, we examined epithelial, mesenchymal, and autophagy markers, including E-cadherin, N-cadherin, vimentin, Snail, P62, Beclin1, and Atg5, using Western blot. HAPSTR1 knockdown caused a significant decline in P62, N-cadherin, vimentin, and Snail expression levels and increased levels of Beclin1, Atg5, and E-cadherin (Figure 2C, 2D). Conversely, HAPSTR1 overexpression resulted in the opposite observations (Figure 2C, 2D). In A2780 cells, overexpression of HAPSTR1 leads to downregulation of P53 and P21 expression levels. (Supplementary Figure 2A). To enhance predictive accuracy, we screened the HAPSTR1-binding proteins identified in IP-MS based on the criterion of having two or more unique peptides. Subsequently, we intersected these results with the binding proteins obtained from published databases (GSE204961) and the BIOGRID website [18], resulting in the identification of 6 proteins as common interacting proteins (Figure 2E). LRPPRC has been reported to influence autophagy and to play a crucial role in promoting proliferation, migration, and invasion in ovarian cancer [16, 17]. Therefore, we explored the relationship between HAPSTR1 and LRPPRC in subsequent sections.

### HAPSTR1 overexpression promotes proliferation, migration, and invasion of OV cells

**Figure 3:**
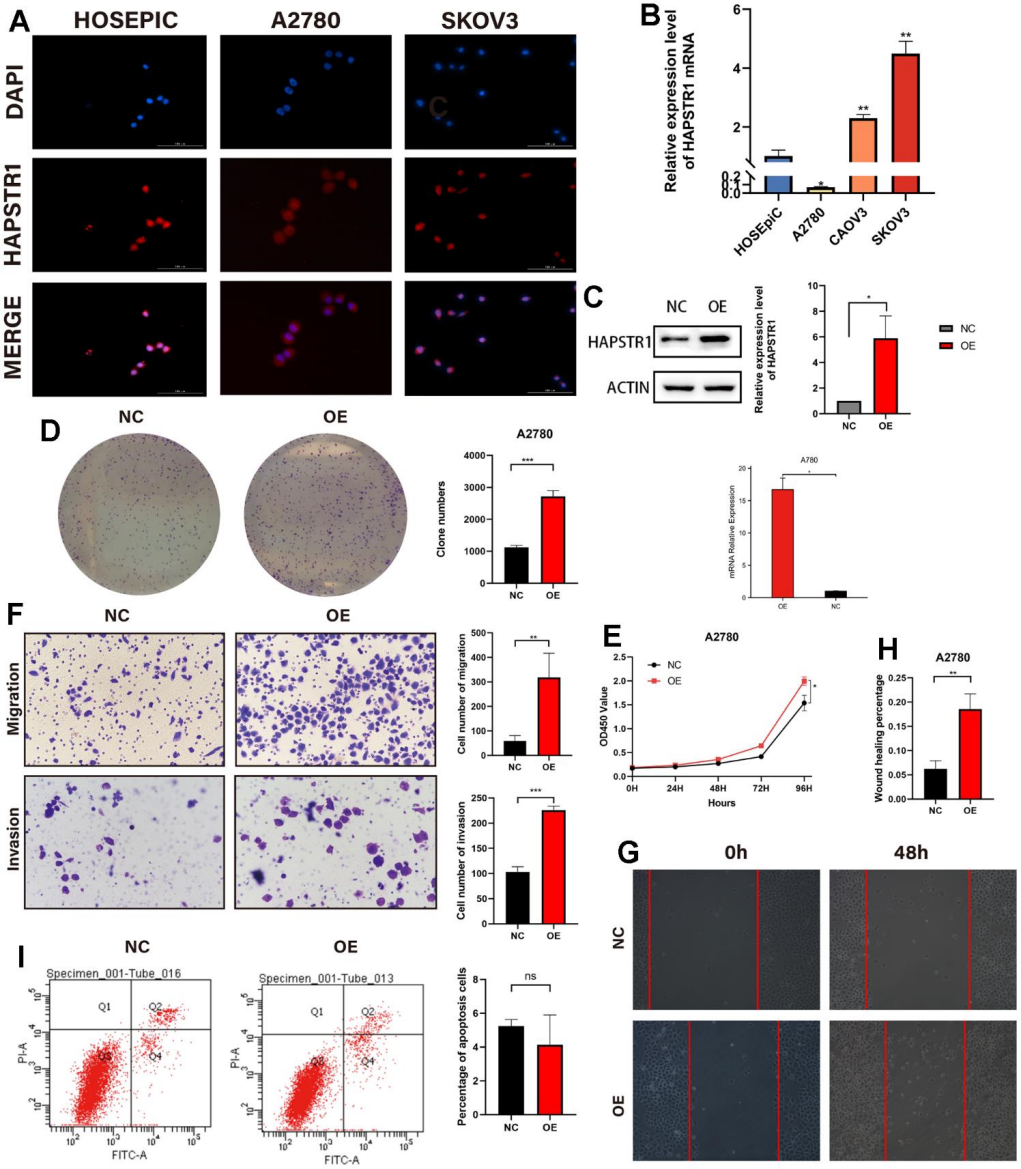
**HAPSTR1 overexpression enhanced proliferation, migration, and invasion of ovarian cancer cells.** (**A**) Cellular localization was visualized in an immunofluorescence staining assay with microscopic observation at 400x. (**B**) The mRNA expression level of HAPSTR1 was detected using real-time PCR. (**C**) Efficiency of HAPSTR1 overexpression was examined using real-time PCR and Western blotting assays. (**D**, **E**) Colony formation and CCK-8 assays were used to show improvements in proliferative capacity induced by HAPSTR1 overexpression. (**F**) The results of the transwell assay showed that HAPSTR1 overexpression enhanced migration and invasion. Original magnification, 200x. (**G**, **H**) A wound healing assay was performed to demonstrate increased migration caused by HAPSTR1 overexpression. (**I**) Flow cytometry was carried out to detect cell apoptosis rates. Each experiment was repeated with three independent replicates. *, *p* < 0.05; **, *p* < 0.01; ***, *p* < 0.001.

To further elucidate the biological functions of HAPSTR1 in OV, the expression level of HAPSTR1 was validated in several types of ovarian cells, and the cellular localization of HAPSTR1 was verified (Figure 3A, 3B). HAPSTR1 was distributed in both the nucleus and cytoplasm, which is consistent with the results of a previous study [9]. The A2780 cell line, which had the lowest HAPSTR1 expression level, was selected for the overexpression experiments. We transfected A2780 cells with the FLAG-HAPSTR1 plasmid (Figure 3C). The results of CCK-8 and colony formation assays showed that the proliferative ability of cells overexpressing HAPSTR1 was stronger than that of NC cells (Figure 3D, 3E). Transwell assays showed that the capacity for migration and invasion substantially increased when HAPSTR1 was overexpressed (Figure 3F). The wound healing assay suggested that HAPSTR1*-*overexpressing A2780 cells exhibited higher migration rates than did NC cells (Figure 3G, 3H). Flow cytometry analysis demonstrated no difference between the percentages of apoptotic HAPSTR1-overexpressing and NC cells (Figure 3I).

### HAPSTR1 knockdown suppressed proliferation, migration, and invasion in cellular model of OV

**Figure 4:**
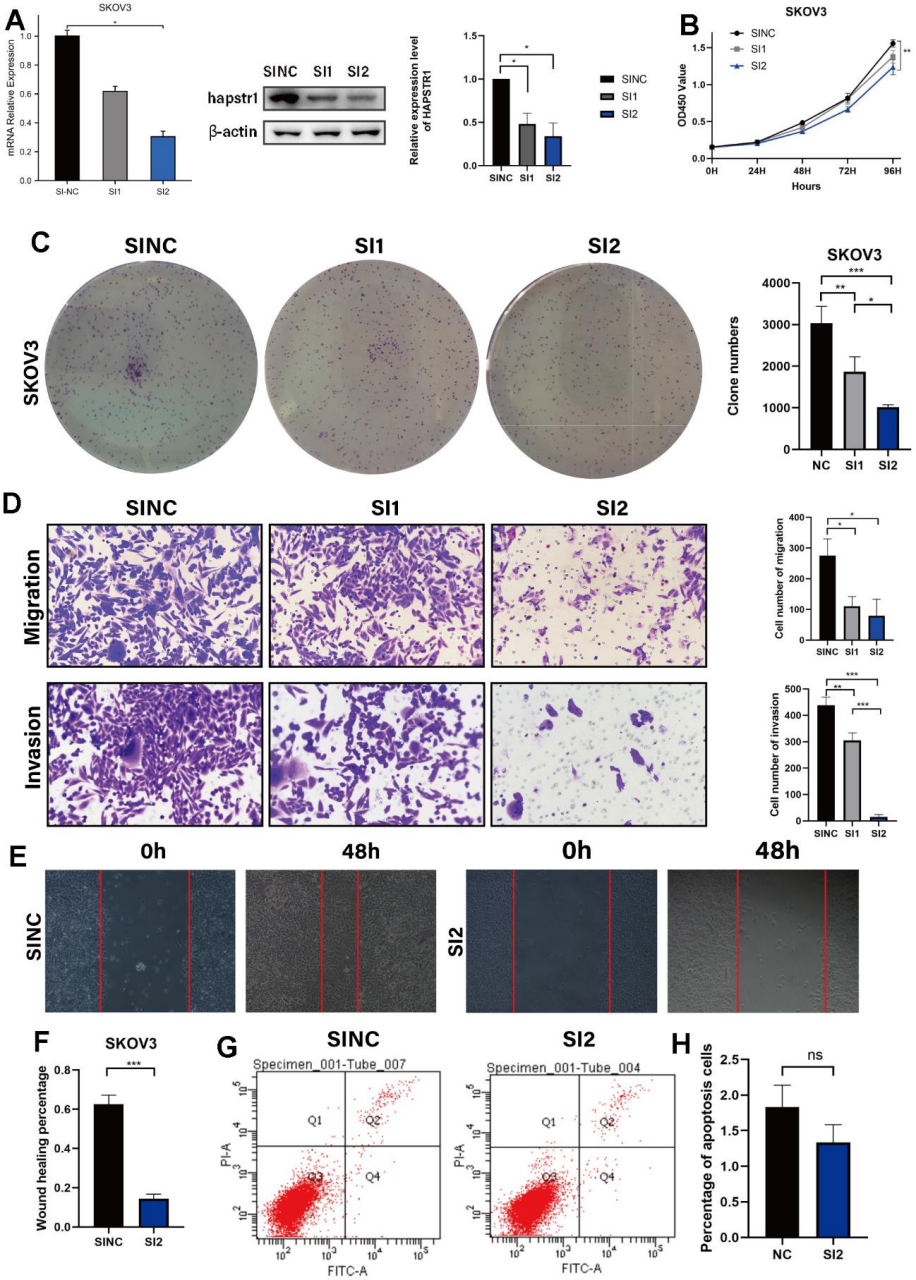
**HAPSTR1 knockdown repressed the proliferation, migration, and invasion capacities of ovarian cancer cells.** (**A**) Efficiency of HAPSTR1 knockdown was examined by real-time PCR and Western blotting assays. (**B**, **C**) The decline in cell viability induced by HAPSTR1 knockdown was detected by colony formation and CCK-8 assays. (**D**–**F**) The results of the transwell and wound healing assays showed that HAPSTR1 knockdown inhibited migration and invasion. Original magnification, 200x. (**G**, **H**) Flow cytometry was carried out to detect cell apoptosis rates. Each experiment was repeated with three independent replicates. *, *p* < 0.05; **, *p* < 0.01; ***, *p* < 0.001.

To further validate the biological function of HAPSTR1 in OV cells, HAPSTR1 was knocked down using two independent siRNAs and verified in SKOV3 cells, which had the highest HAPSTR1 expression (Figure 4A). HAPSTR1 knockdown remarkably inhibited cell proliferation in the CCK-8 and colony formation assays (Figure 4B, 4C). The migratory and invasive capacities of cells were significantly reduced following knockdown (Figure 4D). Moreover, knockdown decreased the wound healing percentage (Figure 4E, 4F). However, the rate of apoptosis did not change significantly (Figure 4G, 4H). Thus, HAPSTR1 knockdown suppressed proliferation, invasion and migration *in vitro*.

### HAPSTR1 supports tumorigenicity *in vivo*

**Figure 5:**
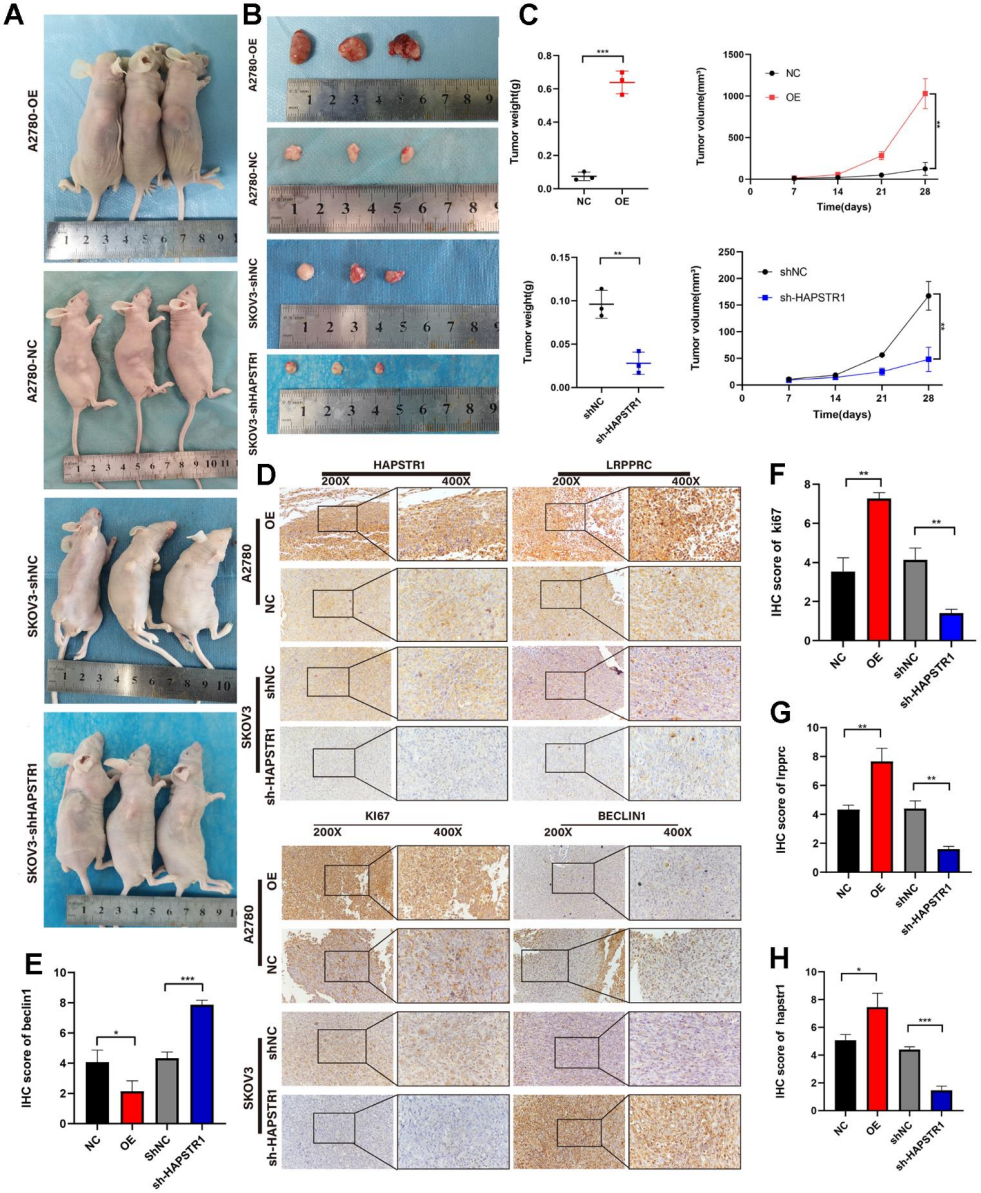
**HAPSTR1 supports tumorigenicity *in vivo* and regulates the EMT pathway and autophagy markers.** (**A**–**C**) Tumor weights and volumes in a subcutaneous xenografted nude mice model demonstrated that HAPSTR1 expression promotes ovarian cancer progression *in vivo*. (**D**–**H**) The immunohistochemistry assay suggested that the HAPSTR1 expression level influenced the expression levels of LRPPRC, KI67, and Beclin1 expression. Original magnification, 200x, 400x. *, *p* < 0.05; **, *p* < 0.01; ***, *p* < 0.001.

To further explore the biological function of HAPSTR1 in mouse models, A2780 cells with stable overexpression of HAPSTR1 were established, and stable SKOV3 cells were transfected with lentiviruses containing the si2-HAPSTR1 sequence. The cells were subcutaneously transplanted to determine the tumorigenicity of OV xenografts in 4-week-old female BALB/c nude mice. The data showed that the hosts of sh-HAPSTR1 cells had smaller tumor volumes and weights than did mice in the shNC group (Figure 5A–5C). Furthermore, HAPSTR1 knockdown markedly reduced LRPPRC and KI67 expression levels and increased Beclin1 expression levels compared to those in control tumors (Figure 5D–5H). Thus, HAPSTR1 knockdown decreased proliferation and enhanced autophagy in OV tumors. HAPSTR1 overexpression resulted in the opposite results.

### HAPSTR1 inhibits ubiquitination of LRPPRC and recruits PSMD14 to interact with LRPPRC

**Figure 6:**
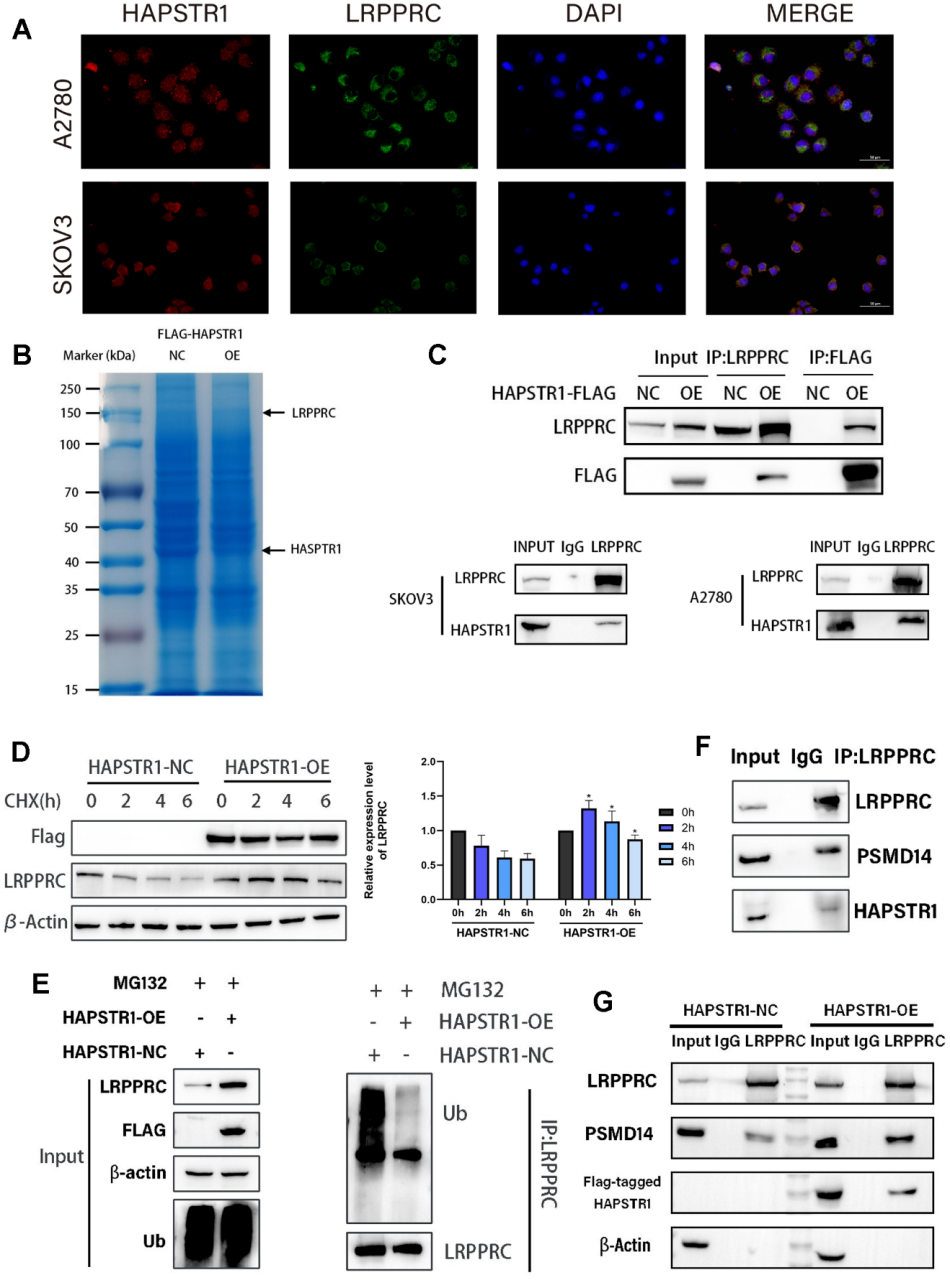
**HAPSTR1 inhibits ubiquitination of LRPPRC and recruits PSMD14 to interact with LRPPRC.** (**A**) Immunofluorescence staining was used to determine the common cellular localization of HAPSTR1 and LRPPRC. Original magnification: 400x. (**B**) The gels in the co-immunoprecipitation (COIP) assay were stained with Coomassie Brilliant Blue. (**C**) A COIP assay was used to detect the interaction between HAPSTR1 and LRPPRC in SKOV3 and A2780 cells. (**D**) HAPSTR promoted protein stability of LRPPRC under cycloheximide treatment. (**E**) HAPSTR1 suppressed ubiquitination of LRPPRC. (**F**) COIP was carried out to detect interactions between LRPPRC and HAPSTR1 and PSMD14 simultaneously. (**G**) HAPSTR1 recruited more PSMD14 for binding to LRPPRC.

Focal adhesion and cadherin-binding processes are closely associated with cell migration and invasion. Autophagy also indirectly affects cancer cell metastasis. Therefore, we investigated the downstream target candidates related to migration and invasion in OV to elucidate the biological functions of HAPSTR1. Additionally, we focused on the role and interactions of LRPPRC. To validate the MS results, immunofluorescence staining was performed to detect the colocalization of HAPSTR1 with LRPPRC. The results showed that co-localization mainly occurred in the cytoplasm (Figure 6A). The gels in the co-immunoprecipitation (COIP) assay were stained with Coomassie Brilliant Blue (Figure 6B). Exogenous and endogenous HAPSTR1 coimmunoprecipitated with LRPPRC, confirming their interaction in SKOV3 cells. Co-immunoprecipitation of LRPPRC with endogenous HAPSTR1 was also performed in A2780 cells (Figure 6C). We speculated that HAPSTR1 was likely to alter the LRPPRC stability because both are ubiquitination-related enzyme substrates [9, 10, 16]. We observed that HAPSTR1 overexpression under cycloheximide treatment relieved LRPPRC degradation compared to that in the control group (Figure 6D). To confirm whether HAPSTR1 affected LRPPRC expression, we performed an LRPPRC ubiquitination experiment, which showed that HAPSTR1 suppressed LRPPRC ubiquitination (Figure 6E). Due to KEGG analysis results showing that the interacting proteins of HAPSTR1 are associated with the ubiquitin-mediated proteolysis pathway, we searched for enzymes reported to play a role in ovarian cancer and are related to LRPPRC and the ubiquitin-proteasome pathway in our IP-MS results [16]. Thus, PSMD14 was identified. We verified that HAPSTR1, LRPPRC, and PSMD14 formed a ternary complex by COIP (Figure 6F). In addition, we observed that more PSMD14 co-immunoprecipitated with LRPPRC when HAPSTR1 was overexpressed (Figure 6G). This suggests that HAPSTR1 promotes the association of LRPPRC with PSMD14. Knockdown of PSMD14 can inhibit the increase in LRPPRC expression induced by HAPSTR1 overexpression (Supplementary Figure 2B).

### HAPSTR1 exerts its oncogenic functions in OV through LRPPRC

**Figure 7:**
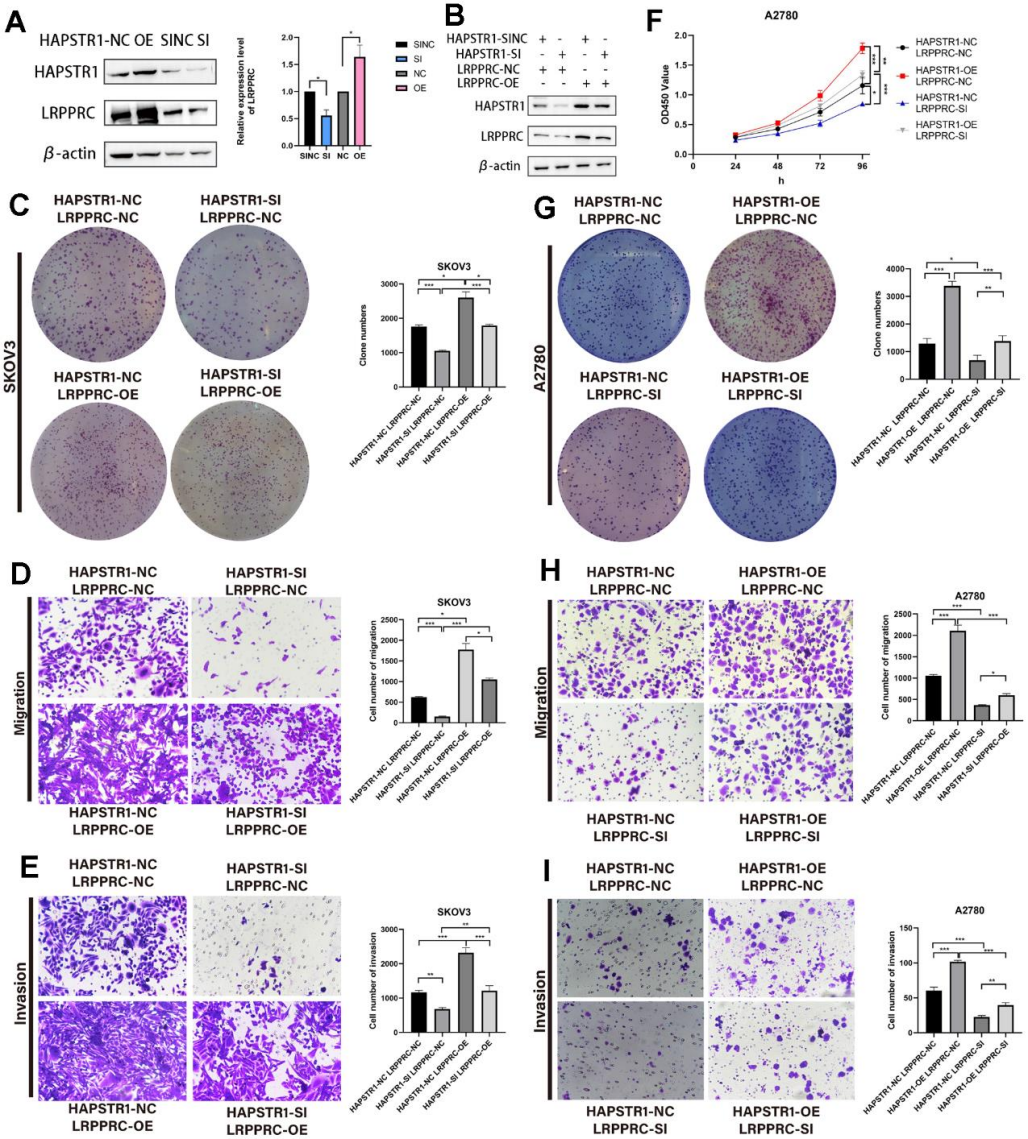
**HAPSTR1 exerts its oncogenic functions in ovarian cancer through LRPPRC.** (**A**) Protein expression levels of LRPPRC were positively correlated with HAPSTR1 via Western blot. (**B**) Transfection efficiency of HAPSTR1 and LRPPRC were examined by Western blot. (**C**–**E**) The colony formation and transwell assays showed that the suppression of proliferative capacity induced by HAPSTR1 knockdown could be rescued by LRPPRC overexpression. Original magnification, 200x. (**F**–**I**) The CCK-8, colony formation, and transwell assays showed that the improvements in proliferative and metastatic capability caused by HAPSTR1 overexpression could be inhibited by LRPPRC knockdown. Original magnification, 200x. Each experiment was repeated with three independent replicates. *, *p* < 0.05; **, *p* < 0.01; ***, *p* < 0.001.

Exploring the regulatory relationship between HAPSTR1 and LRPPRC, we found that HAPSTR1 overexpression led to the upregulation of LRPPRC, whereas HAPSTR1 knockdown exerted the opposite effect (Figure 7A). LRPPRC was suppressed following HAPSTR1 knockdown. We detected the dual transfection efficiency of HAPSTR1 and LRPPRC (Figure 7B). These results demonstrate that LRPPRC overexpression alleviated the suppression of proliferation, invasion, and migration induced by silencing HAPSTR1 in SKOV3 cells (Figure 7C–7E). Subsequently, LRPPRC was knocked down while HAPSTR1 was overexpressed; LRPPRC repression inhibited the proliferation, invasion, and migration caused by HAPSTR1 overexpression in A2780 cells (Figure 7F–7I).

## DISCUSSION

HAPSTR1 stimulates the proliferation and metastasis of some cancer cell lines [9–11]. HAPSTR1 promotes migration in a non-cell autonomous manner via chemokine secretion [9]. In addition, RNA or DNA sequencing of 15 cases of NTRK-rearranged uterine sarcomas revealed that one case showed a fusion of *C16orf72* with *NTRK1* [19]. Further, *HAPSTR1/C16ORF72* was first identified as a target of miR-134, which is associated with tumorigenesis and paclitaxel resistance [8, 20]. In the present study, we confirmed that the mRNA and protein expression levels of HAPSTR1 increased in OV tissues and were significantly related to FIGO stage. Additionally, HAPSTR1 overexpression was correlated to poor clinical outcomes. HAPSTR1 enhanced OV cell proliferation, invasion and migration in a mouse model and *in vitro* model. Furthermore, silencing HAPSTR1 inhibited tumor growth, invasion and migration in a mouse and *in vitro* model. In conclusion, HAPSTR1 was demonstrated to play a critical role in OV progression.

The results of GO/KEGG analysis of HAPSTR1 and its related genes showed that they were closely related to focal adhesion and cadherin-binding processes, which are closely associated with EMT. Disruption of focal adhesions is closely related to EMT, resulting in improved cell migration capability [21–23]. Downregulation of E-cadherin with concomitant upregulation of N-cadherin is a canonical hallmark of EMT in cancer [24–26]. Meanwhile, HAPSTR1 was also a critical molecule in the mitochondrial inner membrane, protein complex, and respiratory chain complex assembly. Mitophagy is activated by inner membrane depolarization, which eliminates dysfunctional mitochondria [27–30]. Thus, we believe that HAPSTR1 affects EMT and autophagy pathways. Our results suggest that HAPSTR1 overexpression promotes EMT and inhibits autophagy markers at the same time. Recent evidence has shown that autophagy is indispensable for cells undergoing EMT to survive migration and dissemination. Autophagy not only orchestrates EMT markers in certain cancers [31], but is also one of the prime mechanisms known to govern EMT [32–35]. Thus, we speculated that HAPSTR1 may stimulate the EMT pathway, which is dependent on autophagy.

Ubiquitination is an important reversible post-translational modification (PTM) in eukaryotic cells [36], and LRPPRC can be regulated by ubiquitination and facilitate ubiquitination. SRA stem-loop-interacting RNA-binding protein (SLIRP) stabilizes LRPPRC by inhibiting ubiquitination and proteasomal degradation [37]. LRPPRC is involved in the enhancement of ASS1 ubiquitination and degradation induced by the TRAF2 E3 ubiquitin ligase [38]. PSMD14 plays an important role in several cancer types as a deubiquitinating enzyme. PSMD14 overexpression is closely related to HNSCC tumorigenesis and mechanically inhibits the ubiquitination and degradation of E2F1 [39]. PSMD14 also enhances hepatocellular carcinoma growth and metastasis by inhibiting GRB2 via deubiquitination [40] and stabilizes LRPPRC via deubiquitination [16]. In the present study, we confirmed that HAPSTR1 interacts and colocalizes with LRPPRC in the cytoplasm. HAPSTR1 inhibited LRPPRC ubiquitination. HAPSTR1 along with PSMD14 promoted LRPPRC expression. HAPSTR1 overexpression stabilizes LRPPRC through PSMD14. In a previous study, HAPSTR1 was necessary for HUWE1 nuclear substrate targeting [10]. Our results showed that HAPSTR1 is also required for PSMD14 substrate capture in the cytoplasm. This indicates that the relationship between HAPSTR1 and ubiquitin-related enzymes is diverse and complex. We also showed that LRPPRC enhanced proliferation, invasion, and migration in OV cells, whereas LRPPRC overexpression rescued the tumor inhibitory effect caused by HAPSTR1 loss-of-function. The results of our rescue experiments showed that LRPPRC participates in HAPSTR1-mediated proliferation invasion and migration. In addition, LRPPRC overexpression enhanced protein expression level of HAPSTR1 in turn. These results illustrate that HAPSTR1 might be a downstream molecule of LRPPRC that forms a positive feedback loop in an autocrine manner. In conclusion, our findings provide new insights into the involvement of HAPSTR1 in ubiquitination and illustrate HAPSTR1 is a novel and valuable target for OV treatment. Because of the ability of LRPPRC to regulate post-translational modification, activate transcription and modulate the m6A modification [41, 42], the mechanisms behind the positive feedback loop require further study. Besides, the effects of PSMD14 and LRPPRC on HAPSTR1 ubiquitination also require further investigation. In our report, HAPSTR1 inhibits autophagy biomarkers, which is inconsistent with the previous report [10]. A possible explanation is HAPSTR1 plays different roles in different cells.

**Figure 8:**
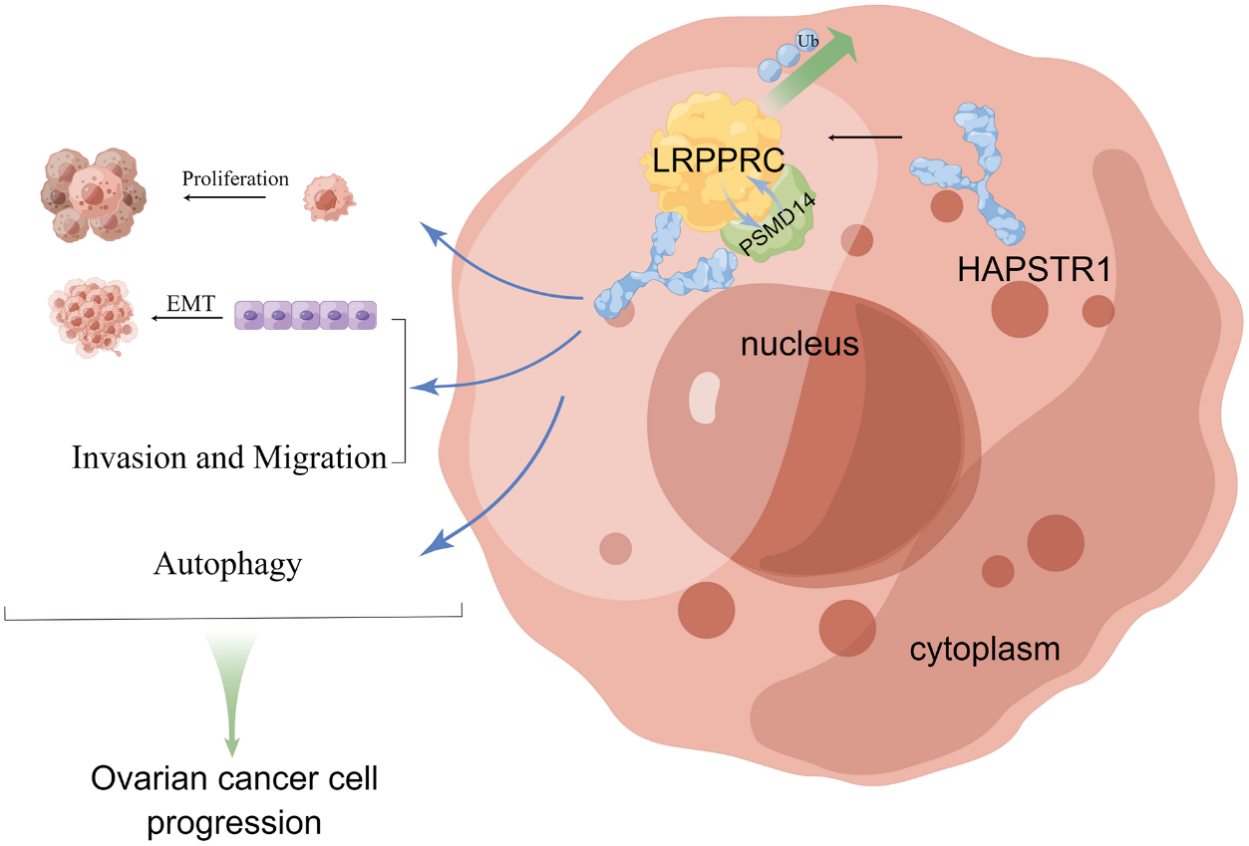
**The schematic diagram of HAPSTR1’s role in promoting the malignant progression of ovarian cancer through recruiting PSMD14 to suppress ubiquitination of LRPPRC.** This figure was drawn with Figdraw (ID:OOYSPcf40f).

In summary, our study showed that HAPSTR1 is overexpressed in OV tissues. This increase in expression was related to early FIGO stage and poor prognosis. Meanwhile, HAPSTR1 activated the EMT signaling pathway and affected autophagy biomarkers. Functionally, HAPSTR1 promoted proliferation, invasion and migration both *in vivo* and *in vitro*. Mechanistically, HAPSTR1 bound to LRPPRC and inhibited its ubiquitination. We also observed a ternary complex comprising HAPSTR1, PSMD14, and LRPPRC, in which HAPSTR1 recruits PSMD14 for interaction with LRPPRC. This demonstrates that HAPSTR1 inhibits LRPPRC degradation through ubiquitination by enhancing the LRPPRC–PSMD14 interaction. Our rescue experiments showed that LRPPRC is involved in HAPSTR1-mediated proliferation, invasion and migration Figure 8. Thus, the mechanistic role of HAPSTR1 in OV is reported for the first time, and our study also has provided new insights into the mechanisms by which LRPPRC influences the malignant progression of ovarian cancer, highlighting LRPPRC as a significant therapeutic target in the context of ovarian cancer treatment.

## MATERIALS AND METHODS

### Patients and specimen collection

Ovarian tissues for real-time PCR and immunohistochemistry (IHC) assays were obtained from patients who underwent surgery at the Shengjing Hospital of China Medical University from 2017 to 2022. No patients received preoperative chemotherapy or radiation therapy. This study was approved by the Research Ethics Committee of China Medical University (2023PS182K).

### Data sources and pre-processing

The gene expression profiles and related clinical characteristics of 381 patients with OV were downloaded from The Cancer Genome Atlas (TCGA). Data from tumor tissue samples were further analyzed by log2 (FPKM+1) transformation. The association between C16orf72 gene expression [ENSG00000182831.12] and clinicopathological parameters was also analyzed by R (4.2.1) ggplot2 [3.3.6], stats [4.2.1], car [3.1-0].

The mRNA expression profile of OV in the Kaplan–Meier plotter (https://kmplot.com/analysis/) [43] was used for survival analysis. The prognostic value of HAPSTR1 mRNA levels was also analyzed. All OV samples obtained from the Kaplan–Meier Plotter were included. Parameters were set as follows: Cohort: Ovarian cancer. Dataset: Combined ovarian cancer datasets including GSE14764, GSE15622, GSE18520, GSE19829, GSE23554, GSE26193, GSE26712, GSE27651, GSE30161, GSE3149, GSE51373, GSE63885, GSE65986, GSE9891 and TCGA. Patient samples: All patients. Probeset: 225183_at. Stratification parameters: Histology: [Only serous histology types included]; Stages: [all stages included]; Treatments: [all treatment types controlled for]; Grades: [all tumor grades included]; TP53 mutation: [Only mutated included].

The survival curves were compared between patient groups with high versus low HAPSTR1 expression, determined by auto select best cutoff. For the probe used in this analysis (225183_at), the cut-off value was set at the expression level of 1962, with an expression range of 788-3410 across the compiled ovarian cancer patients. Statistical significance between the curves was determined by the log-rank test, with *p* < 0.05 considered significant. Hazard ratios with 95% confidence intervals were calculated using Cox proportional hazard regression.

The LinkedOmics database (https://www.linkedomics.org/login.php) [44] was used to obtain HAPSTR1 coexpression profiles with Pearson test. The HiSeq RNA platform and TCGA_OV cohort were selected for the analysis, and the results were visualized as volcano plots and heat maps.

The Metascape (http://metascape.org) [45] online database and R clusterProfiler package [46] were employed for Gene Ontology (GO) function and Kyoto Encyclopedia of Genes and Genomes (KEGG) pathway enrichment analyses of HAPSTR1 co-expressed genes and interacted proteins with HAPSTR1. GO contains biological processes (BPs), cell components (CCs), and molecular functions (MFs), and the signaling pathways were identified by considering both p-value and count number.

TISCH (http://tisch.comp-genomics.org) [47], a tool for single-cell transcriptome analysis in the Gene Expression Omnibus (GEO), was used to explore the relationship between HAPSTR1 and immune cells at the single-cell level.

The TIMER database (http://timer.comp-genomics.org) [48] was used to investigate the role of HAPSTR1 in the OV tumor immune environment.

### Real-time PCR (RT-PCR)

Total RNA was extracted using SevenFast® Total RNA Extraction Kits according to the manufacturer’s instructions (SM132-01; Sevenbio). cDNA was prepared using a PrimeScript RT Reagent Kit according to the manufacturer’s protocol (RR047A; Takara Bio, Kyoto, Japan). TB Green Premix Ex Taq II (Takara Bio) was used for RT-PCR. We used primers synthesized by Sangon (Shanghai, China), and the primer sequences were as follows:

β-ACTIN F’: GGGAAATCGTGCGTGACATTAAG;

β-ACTIN R’: TGTGTTGGCGTACAGGTCTTTG;

HAPSTR1-F: TGGACAATGGTGGAACTAGAAAGCG;

HAPSTR1-R: TCTGTTGCGTTTATGGGTTGGTGAG.

The relative expression of mRNA was normalized to β-ACTIN using the 2−ΔΔCT method.

### Immunohistochemistry

IHC was performed using an immunohistochemistry kit according to the manufacturer’s instructions (E-IR-R211; Elabscience, Wuhan, China). The specimens were scored according to the percentage of positively stained cells (0 = negative; 1 = 1–10%; 2 = 11–50%; 3 >50%) and intensity of staining (0 = no staining; 1 = slight staining; 2 = moderate staining; 3 = strong staining). The final scores were obtained by multiplying the percentage of positive cells by the staining intensity. Specimens with scores 0–3 and 4–9 were assigned to the low-and high-expression groups, respectively. Anti-HAPSTR1 (1:100, OTl2B8; Origene, Rockville, MD, USA), anti-LRPPRC (1:100, 21175-1-AP; Proteintech, Rosemont, IL, USA), anti-Beclin1 (1:100, WL02508; Wanleibio, Shenyang, China), and anti-Ki-67 (1:500, WL0280a; Wanleibio) antibodies were used.

### Cell transfection

Two micrograms plasmid/25 nM siRNA and 6 μL Lipo2000 Transfection Reagent (GLPBIO, Montclair, CA, USA) were added to tubes containing 125 μL serum-free medium, which were then incubated at room temperature for 15 min. The mixed solution was then added to a 6-well plate, and serum-free medium was added for a final volume of 2 mL. After 4–6 h, the transfection medium was replaced with the original culture medium. Full-length cDNA encoding human HAPSTR1 and LRPPRC were amplified by PCR and verified by DNA sequencing. For HAPSTR1 overexpression plasmid production (Flag-HAPSTR1-OE), the sequence of HAPSTR1 (ID: NM_014117) was cloned into the pCDNA3.1-CMV-mcs-3flag-EF1a-puro vectors (blank vector, Flag-HAPSTR1-NC) (Hanbio, Shanghai, China). For LRPPRC overexpression plasmid production (LRPPRC-OE), the sequence of LRPPRC (ID: NM_133259) was cloned into GV712-CMV enhancer-MCS-SV40-puromycin with a HA-tag (blank vector, LRPPRC-NC) (Genechem, Shanghai, China).

The small interfering RNA (siRNA) sequences targeting HAPSTR1 (si1, si2) and the negative control (siNC) were procured from Ribobio (Guangzhou, China). siRNA targeting LRPPRC (LRPPRC-SI) and the negative control (siNC) were obtained from JTSbio (Wuhan, China). Notably, PSMD14 siRNAs (siNC and PSMD14-SI) were also sourced from JTSbio.

### Cell culture

A2780, SKOV3, HosePic, and CAOV3 cells were cultured in RPMI 1640 (Sevenbio, Beijing, China) with 10% fetal bovine serum (Procell, Wuhan, China) and 1% penicillin/streptomycin (Procell). All cell lines were incubated in a humidified incubator at 37° C under 5% CO_2_. Cycloheximide was purchased from GLPBIO. MG132 was purchased from Topscience (Shanghai, China).

### Transwell assay

Eight-micrometer pore chambers (Costar; Corning Inc., Corning, NY, USA) were used for the transwell assays. Fifty thousand cells were plated on the upper transwell chambers containing 200 μL serum-free medium. Matrigel (1:10 dilution) (BD Biosciences, Franklin Lakes, NJ, USA) was added to the upper transwell chambers to detect invasion capability. Additionally, 600 μL RPMI 1640 with 10% FBS was added into the bottom chambers. After culture for 24 h, the membranes were fixed with methanol, stained with 1% crystal violet, and photographed using an inverted microscope (703,548, Nikon, Japan).

### CCK-8 assay

The cell suspension (2000 cells/100 μL) was added to 96-well plates. Ten microliters of CCK-8 reagent (GLPBIO) were added at 24, 48, 72, and 96 h. After 2 hours of incubation at 37° C, cell density was measured at 450 nm (1603301D, Bio Tek, USA).

### Colony formation assay

One thousand cells per well were plated in 6-well plates. After 10 d, the cells were fixed with 4% paraformaldehyde and stained with crystal violet. The number of visible colonies was counted to measure colony formation ability.

### Cell apoptosis assays

The rate of apoptosis was determined by flow cytometry in the dark after staining with an annexin V-FITC apoptosis analysis kit (Sungene Biotech Co., Tianjin, China).

### Wound healing assay

A 200 μL pipette tip was used to scratch straight lines. Cells in the plates were cultured in RPMI 1640 medium without serum for one day. The scratch widths were recorded under a microscope at 0 and 48 h.

### Western blotting

Total protein was extracted using a cell lysis buffer for Western blotting and immunoprecipitation (Beyotime, Shanghai, China) containing PMSF and a protease inhibitor cocktail. Proteins (30 μg/well) were separated using 10% SDS/PAGE and transferred onto PVDF membranes (Millipore, Billerica, MA, USA). The primary antibodies used in this study were against anti-HAPSTR1 (1:1000, OTl2B8; Origene), anti-HAPSTR1 (1:500, OTI2D1; Novusbio, Centennial, CO, USA), anti-LRPPRC (1:5000, 21175-1-AP; Proteintech), anti-β-actin (1:1000, 20536-1-AP; Proteintech), anti-Ub (1:1000, 10201-2-AP; Proteintech), anti-N-cadherin (1:5000, 66219-1-Ig; Proteintech), anti-Beclin1 (1:2000, T55092; Abmart, Berkeley Heights, NJ, USA), anti-P62/SQSTM1 (1:2000, T55546; Abmart), anti-ATG5 (1:2000, T55766; Abmart), anti-vimentin (1:500, WL01960; Wanleibio), anti-snail (1:500, WL01863; Wanleibio), and anti-E-Cadherin (1:500, WL01482; Wanleibio).

### Co-immunoprecipitation (COIP) assay

Exogenous immunocoprecipitation: FLAG-tagged HAPSTR1 overexpressing and negative control cells were resuspended in cleavage solution including cell lysis buffer for Western and IP (Beyotime), Protease Inhibitor Cocktail (EDTA-Free, 100X in DMSO) (Apexbio, Houston, TX, USA) and PSMF (Beyotime). Cell lysis buffer for Western and IP (Beyotime) includes 20mM Tris (pH 7.5), 150mM NaCl, 1% Triton X-100, sodium pyrophosphate, β-glycerophosphate, EDTA, Na3VO4, and leupeptin. Samples were centrifuged at 12,000 rpm for 5 min at 4° C. Protein concentrations were determined by BCA Protein assay (Beyotime). FLAG IP was performed using Anti-Flag Magnetic Beads (Beyotime). The beads were washed three times with TBS. Equal concentrations of each sample (500 μl) were incubated with magnetic beads (20 μl) overnight at 4° C. The next day, beads were washed three times in lysis buffer before elution. 100 μl 3X Flag Peptide solution was added to the beads. The complexes were placed on the rotary mixer at 4°C for 2h. The obtained supernatant was boiled in the loading buffer for 10 min.

For each endogenous immunoprecipitation, 0.5 mL cell cleavage solution was incubated with 4 μg primary antibody or IgG at 4° C overnight. The next day, 20 μl Protein A+G magnetic beads (Beyotime) were added to the complexes, which were then incubated at indoor temperature on the rotary mixer for 1 h. The bead–antibody–antigen complexes were washed three times and boiled in the loading buffer for 10 min.

The retrieved proteins were separated by SDS-PAGE gel, followed by Coomassie Brilliant Blue (Beyotime) staining, and analyzed by mass spectrometry (Genechem, Shanghai, China) on Q Exactive mass spectrometer (Thermo Fisher Scientific, Waltham, MA, USA). In our analysis, proteins identified exclusively in the HAPSTR1 flag-OE group with at least one unique peptide were considered as IP-MS detected proteins.

### Immunofluorescence staining

Prepared cell samples were incubated with anti-HAPSTR1 or anti-LRRPRC primary antibody at 4° C for 12 h and CoraLite594 – conjugated Goat Anti-Rabbit IgG (H+L) (Proteintech) in the dark for 2 h. DAPI (Solarbio, Beijing, China) was used to stain nuclei. Antibodies against LRPPRC (1:100, Proteintech) and C16orf72 (1:100, Proteintech) were used as primary antibodies.

### Animal experiments

The lentiviruses were generated by Hanbio (Shanghai, China). Stably transfected cells were generated. Four-week-old female athymic nude mice were obtained from Huafukang (Beijing, China). The mice were subcutaneously inoculated with a cell suspension (4 × 10^6 cells per mouse) in the dorsal side to establish a tumor-bearing mouse model.

### Statistical analysis

All data were analyzed using SPSS 23.0 (IBM, Armonk, NY, USA) and GraphPad Prism 8.0 (GraphPad Software, La Jolla, CA, USA). Paired sample *t*-tests, unpaired sample *t*-tests, and ANOVA tests were used for comparisons between groups. All results are expressed as mean ± SEM. *p*-values < 0.05 were considered statistically significant.

### Data availability statement

The original data in our study are available from the corresponding author on reasonable request.

## Supplementary Material

Supplementary Figures

Supplementary Table 1

Supplementary Table 2

Supplementary Table 3

## References

[1] Bray F, Ferlay J, Soerjomataram I, Siegel RL, Torre LA, Jemal A. Global cancer statistics 2018: GLOBOCAN estimates of incidence and mortality worldwide for 36 cancers in 185 countries. CA Cancer J Clin. 2018; 68:394–424. 10.3322/caac.21492 30207593

[2] Moufarrij S, Dandapani M, Arthofer E, Gomez S, Srivastava A, Lopez-Acevedo M, Villagra A, Chiappinelli KB. Epigenetic therapy for ovarian cancer: promise and progress. Clin Epigenetics. 2019; 11:7. 10.1186/s13148-018-0602-0 30646939 PMC6334391

[3] Zhang R, Siu MK, Ngan HY, Chan KK. Molecular Biomarkers for the Early Detection of Ovarian Cancer. Int J Mol Sci. 2022; 23:12041. 10.3390/ijms231912041 36233339 PMC9569881

[4] Yousefi M, Dehghani S, Nosrati R, Ghanei M, Salmaninejad A, Rajaie S, Hasanzadeh M, Pasdar A. Current insights into the metastasis of epithelial ovarian cancer - hopes and hurdles. Cell Oncol (Dordr). 2020; 43:515–38. 10.1007/s13402-020-00513-9 32418122 PMC12990730

[5] Tian W, Lei N, Zhou J, Chen M, Guo R, Qin B, Li Y, Chang L. Extracellular vesicles in ovarian cancer chemoresistance, metastasis, and immune evasion. Cell Death Dis. 2022; 13:64. 10.1038/s41419-022-04510-8 35042862 PMC8766448

[6] Chen B, Dragomir MP, Yang C, Li Q, Horst D, Calin GA. Targeting non-coding RNAs to overcome cancer therapy resistance. Signal Transduct Target Ther. 2022; 7:121. 10.1038/s41392-022-00975-3 35418578 PMC9008121

[7] Shuang T, Wang M, Chang S. Hybrid-polymerase chain reaction to identify novel target genes of miR-134 in paclitaxel resistant human ovarian carcinoma cells. Oncol Lett. 2015; 9:2910–6. 10.3892/ol.2015.3137 26137169 PMC4473704

[8] Shuang T, Wang M, Shi C, Zhou Y, Wang D. Down-regulated expression of miR-134 contributes to paclitaxel resistance in human ovarian cancer cells. FEBS Lett. 2015; 589:3154–64. 10.1016/j.febslet.2015.08.047 26363097

[9] Amici DR, Ansel DJ, Metz KA, Smith RS, Phoumyvong CM, Gayatri S, Chamera T, Edwards SL, O’Hara BP, Srivastava S, Brockway S, Takagishi SR, Cho BK, et al. C16orf72/HAPSTR1 is a molecular rheostat in an integrated network of stress response pathways. Proc Natl Acad Sci USA. 2022; 119:e2111262119. 10.1073/pnas.2111262119 35776542 PMC9271168

[10] Monda JK, Ge X, Hunkeler M, Donovan KA, Ma MW, Jin CY, Leonard M, Fischer ES, Bennett EJ. HAPSTR1 localizes HUWE1 to the nucleus to limit stress signaling pathways. Cell Rep. 2023; 42:112496. 10.1016/j.celrep.2023.112496 37167062 PMC10279472

[11] Benslimane Y, Sánchez-Osuna M, Coulombe-Huntington J, Bertomeu T, Henry D, Huard C, Bonneil É, Thibault P, Tyers M, Harrington L. A novel p53 regulator, C16ORF72/TAPR1, buffers against telomerase inhibition. Aging Cell. 2021; 20:e13331. 10.1111/acel.13331 33660365 PMC8045932

[12] Yang D, Sun B, Zhang X, Cheng D, Yu X, Yan L, Li L, An S, Jiang H, Lasorella A, Iavarone A, Zhang S, Zou F, Zhao X. Huwe1 Sustains Normal Ovarian Epithelial Cell Transformation and Tumor Growth through the Histone H1.3-H19 Cascade. Cancer Res. 2017; 77:4773–84. 10.1158/0008-5472.CAN-16-2597 28687618

[13] Das T, Shin SC, Song EJ, Kim EE. Regulation of Deubiquitinating Enzymes by Post-Translational Modifications. Int J Mol Sci. 2020; 21:4028. 10.3390/ijms21114028 32512887 PMC7312083

[14] Spataro V, Buetti-Dinh A. POH1/Rpn11/PSMD14: a journey from basic research in fission yeast to a prognostic marker and a druggable target in cancer cells. Br J Cancer. 2022; 127:788–99. 10.1038/s41416-022-01829-z 35501388 PMC9428165

[15] Sun T, Liu Z, Bi F, Yang Q. Deubiquitinase PSMD14 promotes ovarian cancer progression by decreasing enzymatic activity of PKM2. Mol Oncol. 2021; 15:3639–58. 10.1002/1878-0261.13076 34382324 PMC8637564

[16] Zhao Z, Xu H, Wei Y, Sun L, Song Y. Deubiquitylase PSMD14 inhibits autophagy to promote ovarian cancer progression via stabilization of LRPPRC. Biochim Biophys Acta Mol Basis Dis. 2023; 1869:166594. 10.1016/j.bbadis.2022.166594 36328147

[17] Jiang R, Chen Z, Ni M, Li X, Ying H, Fen J, Wan D, Peng C, Zhou W, Gu L. A traditional gynecological medicine inhibits ovarian cancer progression and eliminates cancer stem cells via the LRPPRC-OXPHOS axis. J Transl Med. 2023; 21:504. 10.1186/s12967-023-04349-3 37496051 PMC10373366

[18] Oughtred R, Rust J, Chang C, Breitkreutz BJ, Stark C, Willems A, Boucher L, Leung G, Kolas N, Zhang F, Dolma S, Coulombe-Huntington J, Chatr-Aryamontri A, et al. The BioGRID database: A comprehensive biomedical resource of curated protein, genetic, and chemical interactions. Protein Sci. 2021; 30:187–200. 10.1002/pro.3978 33070389 PMC7737760

[19] Costigan DC, Nucci MR, Dickson BC, Chang MC, Song S, Sholl LM, Hornick JL, Fletcher CD, Kolin DL. NTRK -Rearranged Uterine Sarcomas: Clinicopathologic Features of 15 Cases, Literature Review, and Risk Stratification. Am J Surg Pathol. 2022; 46:1415–29. 10.1097/PAS.0000000000001929 35713627 PMC9481736

[20] Wu J, Sun Y, Zhang PY, Qian M, Zhang H, Chen X, Ma D, Xu Y, Chen X, Tang KF. The Fra-1-miR-134-SDS22 feedback loop amplifies ERK/JNK signaling and reduces chemosensitivity in ovarian cancer cells. Cell Death Dis. 2016; 7:e2384. 10.1038/cddis.2016.289 27685628 PMC5059884

[21] Cao M, Nie W, Li J, Zhang Y, Yan X, Guan X, Chen X, Zen K, Zhang CY, Jiang X, Hou D. MicroRNA-495 induces breast cancer cell migration by targeting JAM-A. Protein Cell. 2014; 5:862–72. 10.1007/s13238-014-0088-2 25070379 PMC4225486

[22] Lee JM, Dedhar S, Kalluri R, Thompson EW. The epithelial-mesenchymal transition: new insights in signaling, development, and disease. J Cell Biol. 2006; 172:973–81. 10.1083/jcb.200601018 16567498 PMC2063755

[23] Babaei G, Aziz SG, Jaghi NZ. EMT, cancer stem cells and autophagy; The three main axes of metastasis. Biomed Pharmacother. 2021; 133:110909. 10.1016/j.biopha.2020.110909 33227701

[24] Guan X. Cancer metastases: challenges and opportunities. Acta Pharm Sin B. 2015; 5:402–18. 10.1016/j.apsb.2015.07.005 26579471 PMC4629446

[25] Lamouille S, Xu J, Derynck R. Molecular mechanisms of epithelial-mesenchymal transition. Nat Rev Mol Cell Biol. 2014; 15:178–96. 10.1038/nrm3758 24556840 PMC4240281

[26] Hanahan D, Weinberg RA. Hallmarks of cancer: the next generation. Cell. 2011; 144:646–74. 10.1016/j.cell.2011.02.013 21376230

[27] Doblado L, Lueck C, Rey C, Samhan-Arias AK, Prieto I, Stacchiotti A, Monsalve M. Mitophagy in Human Diseases. Int J Mol Sci. 2021; 22:3903. 10.3390/ijms22083903 33918863 PMC8069949

[28] Shen L, Zhan X. Mitochondrial Dysfunction Pathway Alterations Offer Potential Biomarkers and Therapeutic Targets for Ovarian Cancer. Oxid Med Cell Longev. 2022; 2022:5634724. 10.1155/2022/5634724 35498135 PMC9045977

[29] Liao S, Chen L, Song Z, He H. The fate of damaged mitochondrial DNA in the cell. Biochim Biophys Acta Mol Cell Res. 2022; 1869:119233. 10.1016/j.bbamcr.2022.119233 35131372

[30] Yan C, Duanmu X, Zeng L, Liu B, Song Z. Mitochondrial DNA: Distribution, Mutations, and Elimination. Cells. 2019; 8:379. 10.3390/cells8040379 31027297 PMC6523345

[31] Gundamaraju R, Lu W, Paul MK, Jha NK, Gupta PK, Ojha S, Chattopadhyay I, Rao PV, Ghavami S. Autophagy and EMT in cancer and metastasis: Who controls whom? Biochim Biophys Acta Mol Basis Dis. 2022; 1868:166431. 10.1016/j.bbadis.2022.166431 35533903

[32] Nieto MA, Huang RY, Jackson RA, Thiery JP. EMT: 2016. Cell. 2016; 166:21–45. 10.1016/j.cell.2016.06.028 27368099

[33] Damiano V, Spessotto P, Vanin G, Perin T, Maestro R, Santarosa M. The Autophagy Machinery Contributes to E-cadherin Turnover in Breast Cancer. Front Cell Dev Biol. 2020; 8:545. 10.3389/fcell.2020.00545 32714931 PMC7344152

[34] Grassi G, Di Caprio G, Santangelo L, Fimia GM, Cozzolino AM, Komatsu M, Ippolito G, Tripodi M, Alonzi T. Autophagy regulates hepatocyte identity and epithelial-to-mesenchymal and mesenchymal-to-epithelial transitions promoting Snail degradation. Cell Death Dis. 2015; 6:e1880. 10.1038/cddis.2015.249 26355343 PMC4650445

[35] Qiang L, He YY. Autophagy deficiency stabilizes TWIST1 to promote epithelial-mesenchymal transition. Autophagy. 2014; 10:1864–5. 10.4161/auto.32171 25126736 PMC4198370

[36] Gui W, Ott CA, Yang K, Chung JS, Shen S, Zhuang Z. Cell-Permeable Activity-Based Ubiquitin Probes Enable Intracellular Profiling of Human Deubiquitinases. J Am Chem Soc. 2018; 140:12424–33. 10.1021/jacs.8b05147 30240200 PMC8011864

[37] Wei WS, Wang N, Deng MH, Dong P, Liu JY, Xiang Z, Li XD, Li ZY, Liu ZH, Peng YL, Li Z, Jiang LJ, Yao K, et al. LRPPRC regulates redox homeostasis via the circANKHD1/FOXM1 axis to enhance bladder urothelial carcinoma tumorigenesis. Redox Biol. 2021; 48:102201. 10.1016/j.redox.2021.102201 34864630 PMC8645923

[38] Jia H, Yang Y, Li M, Chu Y, Song H, Zhang J, Zhang D, Zhang Q, Xu Y, Wang J, Xu H, Zou X, Peng H, Hou Z. Snail enhances arginine synthesis by inhibiting ubiquitination-mediated degradation of ASS1. EMBO Rep. 2021; 22:e51780. 10.15252/embr.202051780 34184805 PMC8339691

[39] Jing C, Duan Y, Zhou M, Yue K, Zhuo S, Li X, Liu D, Ye B, Lai Q, Li L, Yao X, Wei H, Zhang W, et al. Blockade of deubiquitinating enzyme PSMD14 overcomes chemoresistance in head and neck squamous cell carcinoma by antagonizing E2F1/Akt/SOX2-mediated stemness. Theranostics. 2021; 11:2655–69. 10.7150/thno.48375 33456565 PMC7806466

[40] Lv J, Zhang S, Wu H, Lu J, Lu Y, Wang F, Zhao W, Zhan P, Lu J, Fang Q, Xie C, Yin Z. Deubiquitinase PSMD14 enhances hepatocellular carcinoma growth and metastasis by stabilizing GRB2. Cancer Lett. 2020; 469:22–34. 10.1016/j.canlet.2019.10.025 31634528

[41] Corrêa S, Binato R, Du Rocher B, Ferreira G, Cappelletti P, Soares-Lima S, Pinto LF, Mencalha A, Abdelhay E. ABCB1 regulation through LRPPRC is influenced by the methylation status of the GC -100 box in its promoter. Epigenetics. 2014; 9:1172–83. 10.4161/epi.29675 25089713 PMC4164502

[42] Wang H, Tang A, Cui Y, Gong H, Li H. LRPPRC facilitates tumor progression and immune evasion through upregulation of m6A modification of PD-L1 mRNA in hepatocellular carcinoma. Front Immunol. 2023; 14:1144774. 10.3389/fimmu.2023.1144774 37063837 PMC10097877

[43] Győrffy B. Discovery and ranking of the most robust prognostic biomarkers in serous ovarian cancer. Geroscience. 2023; 45:1889–98. 10.1007/s11357-023-00742-4 36856946 PMC10400493

[44] Vasaikar SV, Straub P, Wang J, Zhang B. LinkedOmics: analyzing multi-omics data within and across 32 cancer types. Nucleic Acids Res. 2018; 46:D956–63. 10.1093/nar/gkx1090 29136207 PMC5753188

[45] Zhou Y, Zhou B, Pache L, Chang M, Khodabakhshi AH, Tanaseichuk O, Benner C, Chanda SK. Metascape provides a biologist-oriented resource for the analysis of systems-level datasets. Nat Commun. 2019; 10:1523. 10.1038/s41467-019-09234-6 30944313 PMC6447622

[46] Yu G, Wang LG, Han Y, He QY. clusterProfiler: an R package for comparing biological themes among gene clusters. OMICS. 2012; 16:284–7. 10.1089/omi.2011.0118 22455463 PMC3339379

[47] Sun D, Wang J, Han Y, Dong X, Ge J, Zheng R, Shi X, Wang B, Li Z, Ren P, Sun L, Yan Y, Zhang P, et al. TISCH: a comprehensive web resource enabling interactive single-cell transcriptome visualization of tumor microenvironment. Nucleic Acids Res. 2021; 49:D1420–30. 10.1093/nar/gkaa1020 33179754 PMC7778907

[48] Li T, Fu J, Zeng Z, Cohen D, Li J, Chen Q, Li B, Liu XS. TIMER2.0 for analysis of tumor-infiltrating immune cells. Nucleic Acids Res. 2020; 48:W509–14. 10.1093/nar/gkaa407 32442275 PMC7319575

