# Supplementary material for: An LRPPRC-HAPSTR1-PSMD14 interaction regulates tumor progression in ovarian cancer
Source: Aging (Albany NY). 2024 Apr 18;16(8):6773–95. doi: 10.18632/aging.205713 (PMC11087107; doi:10.18632/aging.205713)
Supplement: Supplementary Figures [file aging-16-205713-s001.pdf]

SUPPLEMENTARY FIGURES

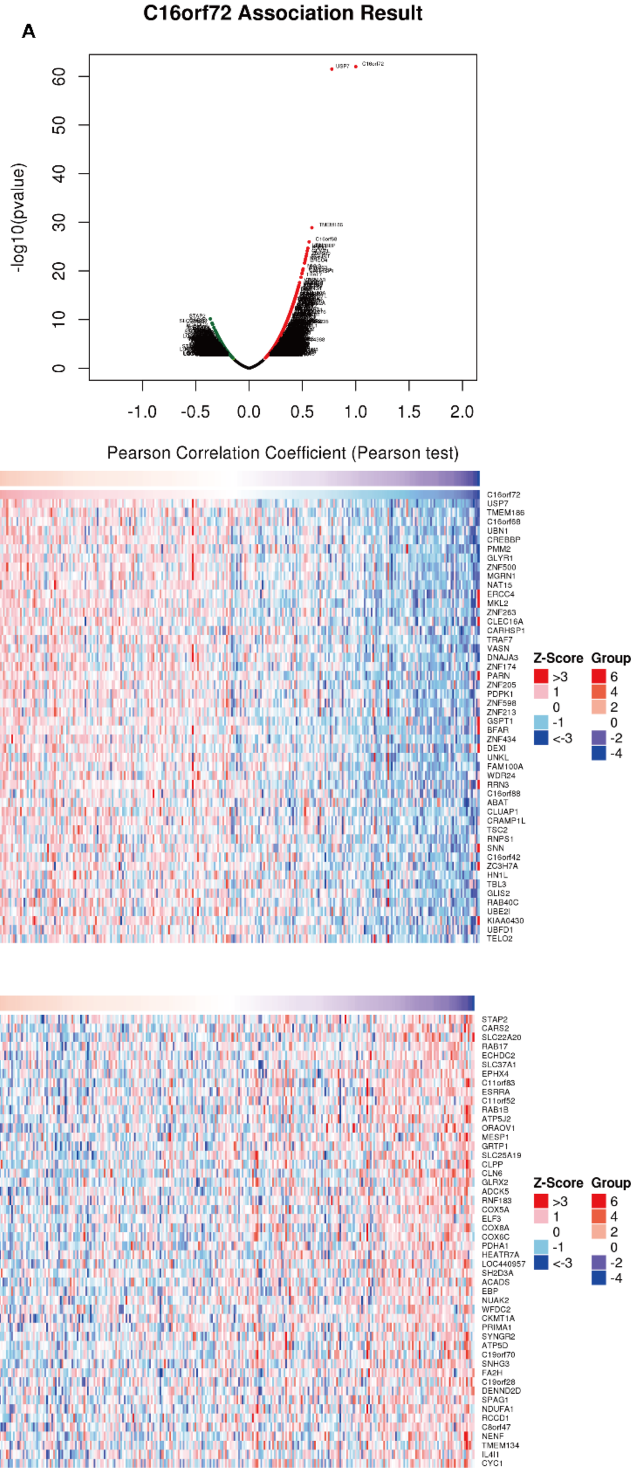

Supplementary Figure 1. Co-expression analysis of HAPSTR1 in ovarian cancer patient data from TCGA using LinkedOmics. (**A**) Volcano plot of genes co-expressed with HAPSTR1. (**B**) A heatmap of the top 50 genes significantly positively or negatively correlated with HAPSTR1 from LinkOmics database analysis.

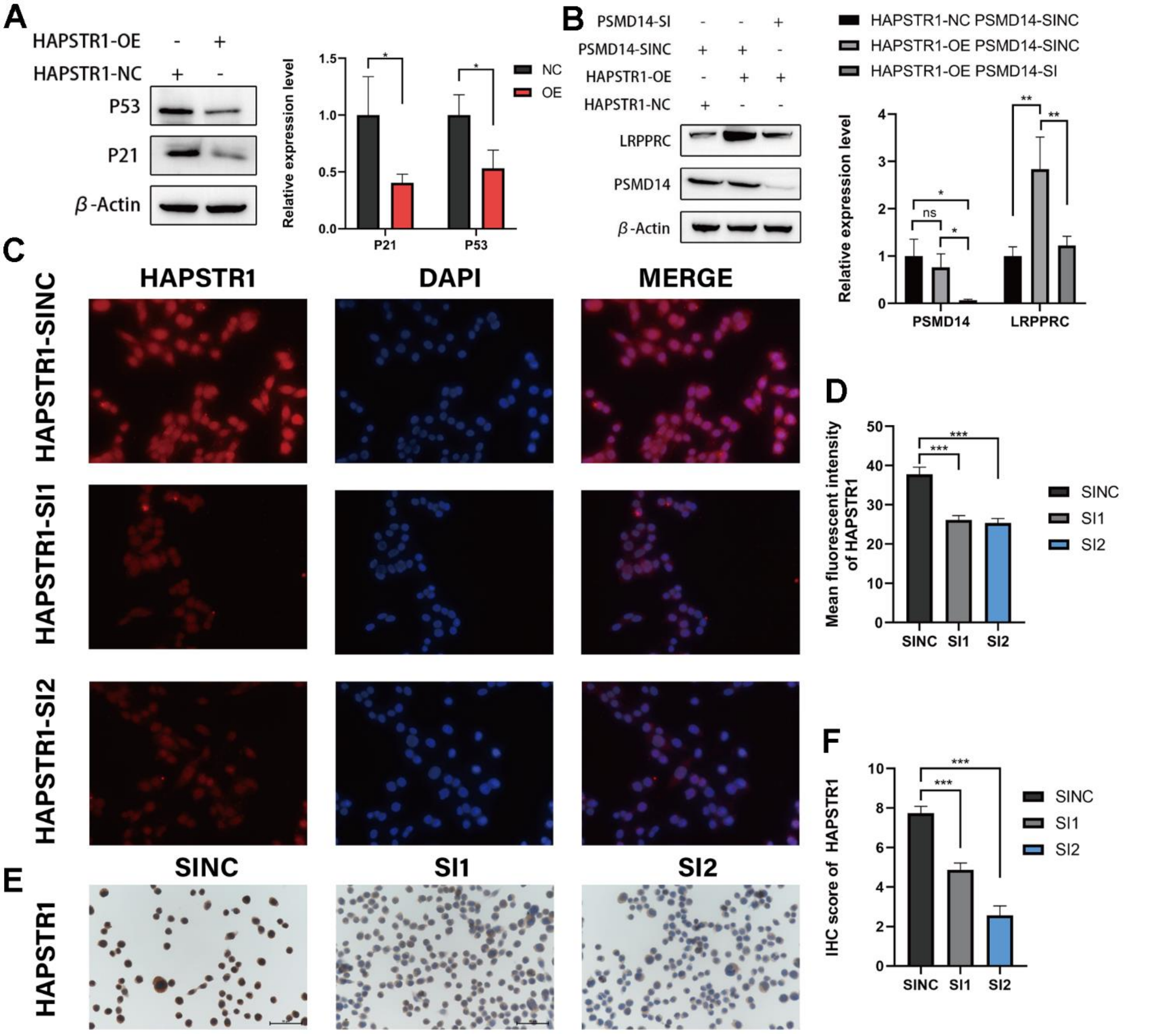

Supplementary Figure 2. Effects of HAPSTR1 overexpression and PSMD14 knockdown on protein expression levels in ovarian cancer cell models. (**A**) Western blot assay was employed to examine the influence of HAPSTR1 overexpression on the expression levels of P53 and P21. (**B**) Western blot assay was utilized to assess the impact of PSMD14 knockdown on the regulation of LRPPRC expression by HAPSTR1. (**C**, **D**) Immunofluorescence assay was conducted to verify the specificity of the primary antibody against HAPSTR1. (**E**, **F**) Immunohistochemistry assay was performed to confirm the specificity of the primary antibody against HAPSTR1.
