# Supplementary material for: An LRPPRC-HAPSTR1-PSMD14 interaction regulates tumor progression in ovarian cancer
Source: Aging (Albany NY). 2024 Apr 18;16(8):6773–95. doi: 10.18632/aging.205713 (PMC11087107; doi:10.18632/aging.205713)
Supplement: Supplementary Table 1 [file aging-16-205713-s002.pdf]

SUPPLEMENTARY TABLE

Supplementary Table 1. Multivariate logistic regression analysis of HAPSTR1 with clinicopathological features in ovarian cancer of TCGA dataset.

Characteristics	Total (N)	OR (95% CI) univariate analysis	P-value univariate analysis	OR (95% CI) multivariate analysis	P-value multivariate analysis
Age	378				
>60	170	Reference			
<=60	208	0.773 (0.515 - 1.161)	0.215		
Anatomic_neoplasm_subdivision	356				
Bilateral	255	Reference			
Right	46	1.462 (0.777 - 2.753)	0.239		
Left	55	1.085 (0.606 - 1.943)	0.784		
Histologic_grade	368				
G3+G4	325	Reference			
G1+G2	43	1.143 (0.604 - 2.162)	0.681		
Race	367				
White	328	Reference		Reference	
Black and yellow	39	2.481 (1.215 - 5.065)	0.013	2.481 (1.215 - 5.065)	0.013
Stage	375				
Stage III+IV	352	Reference			
Stage I+II	23	1.591 (0.671 - 3.772)	0.291		
VeNous_invasion	103				
No	40	Reference			
Yes	63	1.032 (0.467 - 2.280)	0.937		
